# A 24-GHz Front-End Integrated on a Multilayer Cellulose-Based Substrate for Doppler Radar Sensors †

**DOI:** 10.3390/s17092090

**Published:** 2017-09-12

**Authors:** Federico Alimenti, Valentina Palazzi, Chiara Mariotti, Marco Virili, Giulia Orecchini, Stefania Bonafoni, Luca Roselli, Paolo Mezzanotte

**Affiliations:** 1 Department of Engineering, University of Perugia, 06125 Perugia, Italy; federico.alimenti@unipg.it (F.A.); palazzi.valentina.89@gmail.com (V.P.); marco.virili@qorvo.com (M.V.); giulia.orecchini@gmail.com (G.O.); stefania.bonafoni@unipg.it (S.B.); luca.roselli@unipg.it (L.R.); 2 Infineon Technologies Austria AG, Siemensstrasse 2, 9500 Villach, Austria; chiara.mariotti@infineon.com

**Keywords:** Doppler radar sensors, green electronics, all-natural electronic, circuits on cellulose, paper-based substrates, flexible substrates, substrate integrated circuits, Internet of things (IoT)

## Abstract

This paper presents a miniaturized Doppler radar that can be used as a motion sensor for low-cost Internet of things (IoT) applications. For the first time, a radar front-end and its antenna are integrated on a multilayer cellulose-based substrate, built-up by alternating paper, glue and metal layers. The circuit exploits a distributed microstrip structure that is realized using a copper adhesive laminate, so as to obtain a low-loss conductor. The radar operates at 24 GHz and transmits 5 mW of power. The antenna has a gain of 7.4 dBi and features a half power beam-width of 48 degrees. The sensor, that is just the size of a stamp, is able to detect the movement of a walking person up to 10 m in distance, while a minimum speed of 50 mm/s up to 3 m is clearly measured. Beyond this specific result, the present paper demonstrates that the attractive features of cellulose, including ultra-low cost and eco-friendliness (i.e., recyclability and biodegradability), can even be exploited for the realization of future high-frequency hardware. This opens opens the door to the implementation on cellulose of devices and systems which make up the “sensing layer” at the base of the IoT ecosystem.

## 1. Introduction

In recent years, Internet of things (IoT) has been driving academic and industrial research [1,2]. According to the IoT vision, objects are able to gather information directly form the surrounding environment, to communicate among them by means of machine-to-machine (M2M) protocols, and address this information to the Internet without any human intermediation: the “smart object” concept has been coined. It is evident that the electronic hardware of these smart objects has to be conceived starting from radically new paradigms. First, in view of the massive deployment of smart objects, ultra-low cost fabrication technologies of on-board electronics have to be pursued in order to minimize the economic impact of the added smart capabilities and hopefully maintain it negligible. Second, the electronics added to provide everyday items with autonomous sensing and communication capabilities have to be consistent with their life cycle. This means that the hosted electronics must be biodegradable or recyclable.

An impressive datum concerns the latter point: the United Nations estimates that humans throw away about 50 million metric tons of electronics every year [3] and this number will surely increase in the IoT era. The pollution associated with the electronic waste is mainly due to printed circuit boards (PCB). Glass-reinforced substrate materials such as FR4 are dangerous because of their dust and because they contain brominated bisPhenol-A (BPA) epoxy resins. One way to address such a problem is to substitute standard PCB materials with bio-degradable materials such as, for instance, cellulose-based composites [4]. The use of these composites have already been demonstrated even at microwave frequencies [5,6,7,8,9]. Up to now, however, only very simple radio frequency (RF) building blocks, mainly antennas, are available on cellulose. This contribution focuses on these hardware implications, discussing, as a case of study, a typical microwave-wave sensor, implemented according to the philosophy proposed by Savage [3]. Without lack of generality, we consider a motion radar sensor for low-cost IoT applications. The detection of motion, indeed, is a well-known problem with applications ranging from safety (detection of dangerous moving objects), security (anti-theft systems) and health-care (detection of the heartbeat and breathing rates) [10,11], to building automation (intelligent solid-state lighting systems), traffic monitoring [12,13] and robot control. A review of recent technical advances in Doppler radars for healthcare applications can be found in [14], and the basics of microwave doppler radar systems for biomedical applications are investigated in [15]. The signal reflected for instance by the chest and heart of a person contains information about cardiac, respiratory, and arterial movements [15,16,17], as well as about blood flow velocity in major vessels [15]. Electromagnetic (em) waves, indeed have advantages with respect to ultrasonic medical imaging: ultrasonic sensing requires contact of the source and receiver with the human body, whilst the penetration capability of non-metallic objects by em signals allows for contactless and non-invasive monitoring [16]. Radar technologies can also be used in wireless sensor networks to collect spatio-temporal data from animals and to model their movement behavior [18,19]. In this perspective, one can observe that the Doppler radar has the advantage of providing a completely automatic activity recognition system. It can penetrate barriers which obscure optical systems and it operates over long distances, regardless of whether it is day- or night-time. Another very interesting application is the measurement of surface-flow velocity of rivers for the environmental monitoring.

Radar sensors have evolved from bulky waveguide structures [20] to single-chip solutions, the latter based on silicon Complementary Metal-Oxide Semiconductors (CMOS) [21], Silicon-Germanium (SiGe) Bipolar-Complementary Metal-Oxide Semiconductors (BiCMOS) [22] or III-V [23,24] processes. These sensors rely on Doppler [25,26,27] or Frequency-Modulated Continuous Wave (FMCW) radar architectures [28,29]. Finally, several products have been placed on the market [29,30]. All these designs use standard electronic technologies, mostly based on planar circuits and glass-reinforced PCBs. Recently a FMCW ground penetrating radar (GPR) front-end was designed on a paper substrate, but it operates in the Very High Frequency (VHF) – Ultra High Frequency (UHF) bands frequency range [31].

In this contribution, a Doppler radar sensor for motion detection is proposed. In particular, the whole radar front-end and its antenna are fabricated on cellulose and are experimentally characterized. The originality of the proposed solution with respect to a previous work by our group [32] lies in the fact that for the first time, a multilayer cellulose substrate is used to integrate the whole front-end in a very compact size (comparable to that of a postage stamp). Furthermore, the radar is modeled in a theoretical way: the intrinsic front-end sensitivity γ is introduced and verified against experiments. The multilayer substrate is composed by two photo-paper sheets glued together, the ground plane being in the middle, the antenna and the electronic circuitry being on the top and bottom faces, respectively. The metal tracks are realized by utilizing a copper adhesive laminate in order to have a low-loss conductor [33]. The radar sensor works at 24 GHz, a record frequency for circuits on cellulose with such complexity, and is validated considering real-case applications. The prototyped front-end uses an external voltage-controlled oscillator (VCO), although an effort to build an oscillator on cellulose is ongoing. At the moment the main difficulties are represented by the limited Q-factor of paper-based resonators and by the package parasitics of available transistors.

The fact that such a complex system performs as expected, according to the performance of the single building blocks, testifies to the robustness of the proposed technology. A campaign of measurements has been carried out to analyze and characterize the proposed front-end. The front-end operation is analyzed from both a theoretical and an experimental point of view. Through this study we prove that green electronic systems are capable of operating up to the boundary between microwaves and millimeter-waves, providing recyclable and ultra-low cost solutions for the electronic hardware of the upcoming era.

## 2. Basic Theory and Front-End Sensitivity

**Figure 1:**
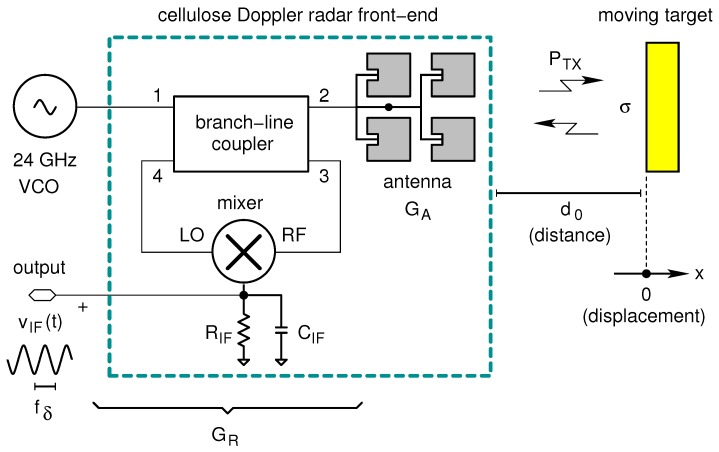
Block diagram of the radar sensor. The circuit uses a branch-line power divider to couple the voltage-controlled oscillator (VCO) to both the antenna and the mixer. If the target moves, a Doppler frequency shift $f_{\delta }$ is produced in the reflected wave and is detected by the mixer circuit. The resistance $R_{IF}$ and the capacitor $C_{IF}$ constitute a low-pass filter and terminate the mixer output port. LO: local oscillator; RF: radio frequency.

The sensor architecture is shown in Figure 1 and is based on the Doppler radar proposed by Catena et al. [34]. It consists of an external oscillator operating at 24 GHz, a single-balanced diode mixer, a branch-line coupler and a planar patch array antenna. The last three blocks are fabricated on cellulose.

In Figure 1 the signal generated by the external voltage-controlled oscillator (VCO) is equally divided into two parts by using a hybrid branch-line coupler: the first half of the signal feeds the antenna (transmitting path), while the second half feeds the local oscillator (LO) port of the mixer. The signal coming from the antenna is sent to the radio frequency (RF) port of the mixer (receiving path) and, thereon, to the oscillator. The latter is obviously an unwanted signal but, thanks to the low power level of the received wave, it does not affect the oscillator operation. The separation between transmitter and receiver is guaranteed by the isolation parameter of the quadrature hybrid (branch-line coupler); the isolation is around 20dB for the implemented circuit. Such a solution, although non-optimal (there are at least 3dB of losses in the receiving path), allows us to avoid the usage of expensive and bulky components such as circulators and can be realized with a fully planar geometry. A mathematical treatment of the leakage between transmitting (TX) and receiving (RX) channels of a Doppler radar can be found in [25].

A detailed theoretical description of Doppler radar motion sensors is given by Droitcour et al. [10,35] and will not repeated here. The Droitcour papers also explain the range correlation concept, i.e., the basic mechanism that allows a small Doppler shift (down to a few Hz) to be detected on top of a microwave carrier (24 GHz in our case) that is affected by the oscillator phase noise. According to these studies, the output signal $v_{IF}\left(t\right)$ of a Doppler radar can be modeled, in the time domain, as follows:

(1) $$v_{IF}\left(t\right)=\alpha \;\cos\;\left[\theta +\frac{4\;\pi \;x(t)}{\lambda }+\Delta \phi \left(t\right)\right]$$

(2) $$\alpha =\sqrt{2\;G_{RX}\;\frac{P_{TX}\;\lambda^{2}\;G_{A}^{2}\;\sigma }{{\left(4\;\pi \right)}^{3}\;d_{0}^{4}}\;R_{IF}\;}$$

(3) $$\theta =\frac{4\;\pi \;d_{0}}{\lambda }+\theta_{0}$$

In these equations $d_{0}$ is the target distance at t=0; x(t) is the time varying target displacement, which is assumed to be much lower than $d_{0}$; Δϕ(t) is the residual phase noise; λ is the carrier wavelength; and $\theta_{0}$ is the phase offset. The remaining quantities can be defined with reference to Figure 1 and are related to the front end. In particular: $P_{TX}$ is the transmitted power; $G_{A}$ is the antenna gain (with respect to the isotropic radiator); $G_{RX}$ is the available power gain of the whole front-end; $R_{IF}$ is the mixer termination resistance (i.e., where the output voltage is developed); and σ is the radar cross-section of the target. The scale constant of the front-end, α, is estimated using the radar equation in a way similar to what done by [35,36].

The output of the front-end is the IF voltage $v_{IF}$; as already mentioned it is obtained by mixing the received voltage signal with the LO signal and low-pass filtering the mixer output. Such a filtering action is accomplished by the capacitor $C_{IF}$.

The front-end in Figure 1 can work in two modes: in Doppler radar and Doppler vibrometer mode [37]. In the first case, (Doppler radar) the displacement x(t) can be expressed as:

(4) x(t)=vt

where *v* is the relative speed of the moving target with respect to the radar, more specifically, the target velocity component along the line connecting the target itself with the radar antenna. This implies that the second term of the argument in (1) can be written as:

(5) $$\frac{4\;\pi \;x(t)}{\lambda }=2\;\pi \;f_{\delta }\;t$$

$f_{\delta }$ being the well-known Doppler frequency:

(6) $$f_{\delta }=2\;f_{0}\;\frac{v}{c_{0}}$$

where $f_{0}$ is the carrier frequency (24GHz in our case) and $c_{0}$ is the speed of light in a vacuum (about $3\times 10^{8}\;m/s$). As a result (1) becomes:

(7) $$v_{IF}\left(t\right)=\alpha \;\cos\;\left[2\;\pi \;f_{\delta }\;t+\theta +\Delta \phi \left(t\right)\right]$$

Computing the quantity $2f_{0}/c_{0}$, a Doppler frequency of about 160Hz per m/s of relative speed is estimated. This means that, when an object is moving, the Doppler radar sensor can detect its speed and thus its presence.

On the other hand, when θ is an odd multiple of π/2 and x(t)≪λ, the radar works as a Doppler vibrometer, as illustrated in [10]. Under this condition, the cosine function in (1) can be approximated by its argument:

(8) $$v_{IF}\left(t\right)\approx \alpha \;\left[\frac{4\;\pi \;x(t)}{\lambda }+\Delta \phi \left(t\right)\right]$$

This is a significant result showing that the output signal is proportional to the displacement x(t) summed to the residual phase-noise Δϕ(t) and that the front-end can be used as a contactless vibrometer, i.e., a scientific instrument capable of measuring the amplitude and frequency of a mechanical vibration. When θ in (1) is an integer multiple of π, instead, the modulation sensitivity is decreased and the system no longer works as a vibrometer, [10]. These null points occur with a periodicity equal to λ/4 of the radar-to-target distance $d_{0}$; i.e., every 3.1mm at 24GHz. As a final consideration, it is important to note that the same scale constant α can be used to model the output voltage of the front-end $v_{IF}$ in both operation modes. Such a constant can be written as the product of the intrinsic front-end sensitivity γ times a factor accounting for the distance $\left(\lambda \sqrt{\sigma }/d_{0}^{2}\right)$:

(9) $$\alpha =\gamma \;\left(\frac{\sqrt{\sigma }\;\lambda }{d_{0}^{2}}\right)$$

(10) $$\gamma =\sqrt{2\;\frac{P_{TX}\;G_{A}^{2}\;G_{RX}}{{\left(4\;\pi \right)}^{3}}\;R_{IF}}$$

On the basis of the previous equations one can estimate the instrinsic sensitivity of the cellulose-based front-end. It operates at $f_{0}$ = 24 GHz (λ = 12.5 mm) with a transmitted power of $P_{TX}$ = 5 mW (7dBm). Assuming an antenna gain $G_{A}$ = 7.4 dBi (four patch array) an overall front-end gain $G_{RX}$ = −14 dB (passive receiver) and a mixer termination resistance $R_{IF}$ = 240 Ω, γ≈ 38.2 mV is obtained. More details about these system parameters can be found in the Appendix A, where the building-blocks of the radar sensor are briefly discussed.

## 3. Materials and Methods

The 24-GHz front-end is manufactured using the adhesive copper-laminate technology described by Alimenti et al. [33,38]. This technology relies on a copper adhesive tape shaped by a photo-lithographic process and then transferred to the cellulose substrate by means of a sacrificial layer. Photo-paper by Mitsubishi Electric is adopted as a substrate material in all prototypes. The multilayer structure is composed by two cellulose-based substrates and three metal layers, as described below. The dielectric characteristic of the substrate was determined by evaluating the phase delay of microstrip lines up to 30 GHz, as reported in [39].

### 3.1. Adhesive Copper-Laminate Process

**Figure 2:**
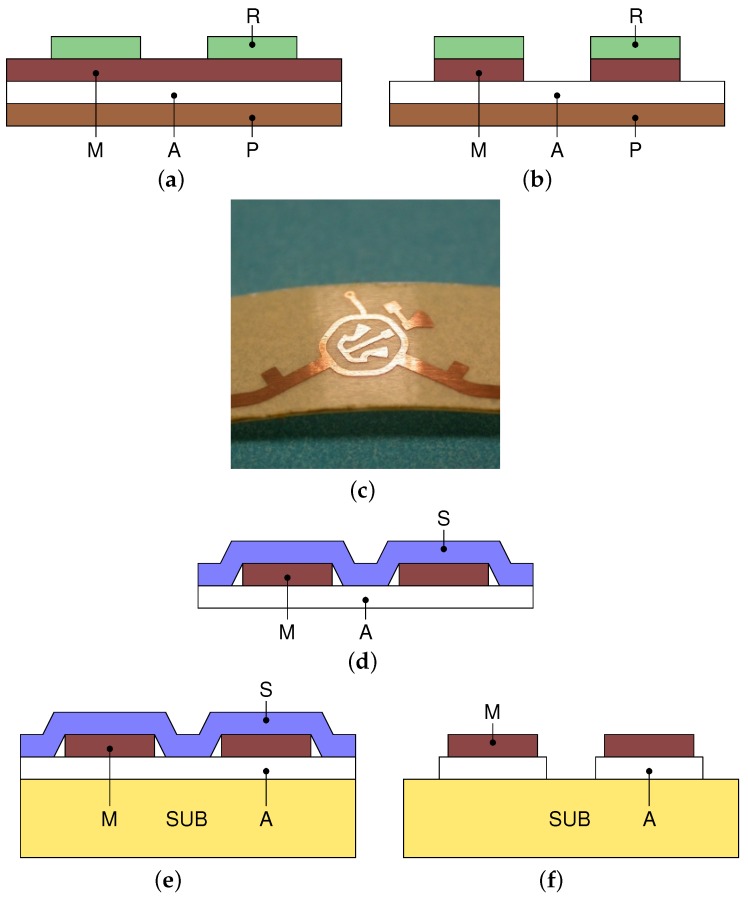
Fabrication process. (**a**) Photo-resist deposited on the metal layer and patterned using a mask, UV and a developer. (**b**) Wet etching of the metal surface. (**c**) Adhesive laminate after the etching of the metal layer: the adhesive material underneath is exposed. (**d**) Application of the sacrificial layer and removal of the protection layer. (**e**) Circuit transferred to the host substrate. (**f**) Sacrificial layer removal; the last step also removes the adhesive material. M: metal, A: adhesive, P: protection, R: photo-resist, S: sacrificial layer, SUB: host substrate.

The process is illustrated in Figure 2. First, a photo-resist film is deposited on the metal surface of an adhesive copper laminate. Using a standard photo-lithography approach, such a film is patterned by means of a mask with the circuit shape, an UV light source and a developer (a NaOH solution with pH 12) to remove the unimpressed film (see Figure 2a). Then, the metal surface is etched by means of ferric chloride as in Figure 2b,c. After etching, the adhesive material is exposed at the metal side while it remains protected on the other side. At this point a sacrificial layer is applied on the metal surface and then the protection layer is removed (see Figure 2d). The sacrificial layer allows keeping the relative distances among the circuit traces, although they are not physically connected. In our case the sacrificial layer is a paper-adhesive tape. The adhesive of the sacrificial layer must be less strong than the glue of the copper tape itself to avoid accidental trace removal. As final steps, the etched metal is transferred to the hosting substrate (Figure 2e), and finally, the sacrificial layer is removed, Figure 2f. The last action also removes the exposed adhesive material.

### 3.2. Fabrication of The Multilayer Cellulose-Based Front-End

**Figure 3:**
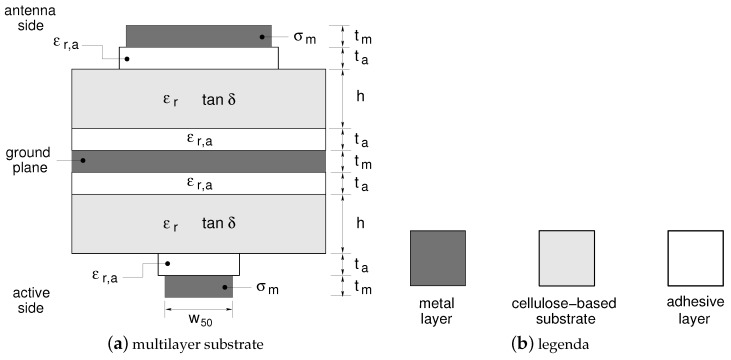
Multilayer substrate. (**a**) Cross-section of the multilayer substrate structure adopted for the fabrication of the 24-GHz radar front-end. (**b**) Materials. Bulk copper with conductivity $\sigma_{m}=5.8\times 10^{7}\;S/m$ is adopted to implement all the metal layers. The substrate parameters as follows: h=230μm, $t_{a}=30\;\mu m$, $t_{m}=35\;\mu m$. The photo-paper and the acrylic adhesive relative permittivity are: $\varepsilon_{r}=2.9$ and $\varepsilon_{r,a}=1.3$ respectively. The photo-paper loss tangent is: tanδ=0.08.

In order to reduce the occupied substrate area, a completely new front-end with respect to [32] is implemented. It adopts the multilayer substrate reported in Figure 3a. In this geometry the top layer is devoted to the antenna array, while the bottom layer hosts the remaining front-end circuitry (mixer and branch-line coupler). The top and the bottom metal layers share a common ground plane located in the middle of the structure. The top (antenna) and bottom (active circuitry) layers are connected by a via-through fabricated with a copper wire of 190μm in diameter. To allow for such a connection without any signal short-circuit, a circular portion of the metal surrounding the via is etched from the ground plane. The main substrate parameters are quoted in the caption of Figure 3. The frequency response of such an interconnection was carefully optimized via electromagnetic simulation (see Appendix A).

### 3.3. Building-Block Design and Characterization

The proposed 24-GHz radar front-end is a complete system operating at quite a high frequency. To ensure that it works correctly, both an accurate design and characterization methodology are adopted. First of all, the front-end is divided into four basic building blocks, namely: a patch array antenna (to transmit and receive the radar signal); a mixer (to extract the Doppler frequency component); a branch-line coupler (to couple together the antenna, VCO and mixer) and a via-through interconnection (to connect both sides of the multilayer cellulose-based circuit). Secondly, each of the above building blocks is designed as a separate circuit. The design activity relies on basic design formulas, circuit simulations simulations and, when needed, 3D electromagnetic numerical analyses. The latter methodology is particularly relevant to optimize all the distributed components of the radar front-end such as the antenna, the branch-line coupler, the via-through interconnection and all the microstrip transmission lines. Finally, as a third step, the building blocks are fabricated on cellulose as stand-alone circuits and experimentally characterized. The measured frequency responses and figure of merit (e.g., the conversion loss of the mixer) are carefully compared with the simulated performance confirming, in all cases, the correctness of the design. The design and measurement of the main building blocks, namely: antenna, branch-line coupler and mixer, are provided in the Appendix A.

## 4. Results

**Figure 4:**
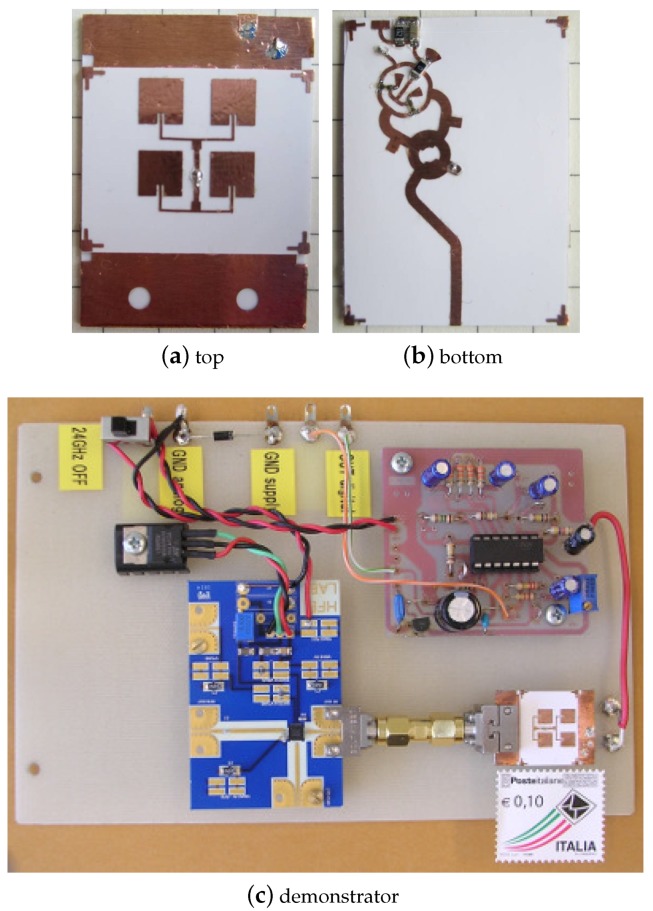
Fabricated 24-GHz front-end on a multi-layer cellulose-based substrate. (**a**) Antenna side. (**b**) Active circuit side. (**c**) Demonstrator including external VCO, intermediate frequency (IF) amplification and triggering stages. The used substrate area is $20\times 27\;{\mathrm{mm}}^{2}$. The realized cellulose circuit has the size of a postage stamp.

The implemented front-end is shown in Figure 4. First, the antenna is etched from the Cu laminate and transferred to one sheet of photo paper (see Figure 4a). Then, with the same approach, the microwave circuitry is realized and attached to the second substrate (see Figure 4b). Finally the ground plane is positioned between the two substrates (i.e., the antenna and the microwave circuit substrates) and everything is glued together. Note that, during the last step, all the pieces are aligned by means of an optical instrument. A small hole (800μm in diameter) is previously etched on the ground plane to allow for a via-through connection (wire measuring 190μm in diameter) between the antenna and the other circuit components.

Once the multilayer board is prepared, two beam-lead Schottky diodes are soldered to the circuit along with the other few components of the front-end: a 0Ω resistor used as a jumper, a 240Ω resistor representing the intermediate frequency (IF) load of the mixer ($R_{IF}$) and a 10-nF filtering capacitor at the IF output ($C_{IF}$). The antenna has a gain of 7.4 dBi with a 48-degree half-power beam-width [40]. The front-end is 20mm wide and 27mm high, resulting in an overall size comparable to that of a postage stamp.

In order to verify the Doppler radar operation, the demonstrator of Figure 4c is implemented. It is composed of a voltage-controlled oscillator (VCO) based on the Hittite HMC739LP4 (Analog Devices, Norwood, MA, USA) integrated circuit. Such an oscillator provides the 24-GHz carrier at the front-end with an available power of about 11dBm (see the device data sheet). Note that the transmitted power is less than 11dBm because of the branch-line coupler insertion losses; these are equal to about 4dB. As a result the transmitted power $P_{TX}$ is around 7dBm (i.e., 5mW in linear scale).

The oscillator is connected to the cellulose-based front-end by means of two Southwest coaxial to microstrip launchers. Then, the Doppler signal coming out of the IF port of the mixer, is sent to a low frequency (LF) amplifying and triggering unit. This unit is based on a low-cost operational amplifier (OPA) and has an amplifying chain formed by three inverting stages in cascade. These stages are with AC coupled to the front-end to produce a high-pass filter with a cut-off frequency of 1Hz. The overall LF gain at mid-band is about 67dB and can be further increased to improve the range of the radar sensor. The amplifier chain is followed by an inverting Schmitt’s trigger with adjustable thresholds. Such a trigger is used to provide a digital output that can be acquired by a micro-controller unit.

### 4.1. People Detection

**Figure 5:**
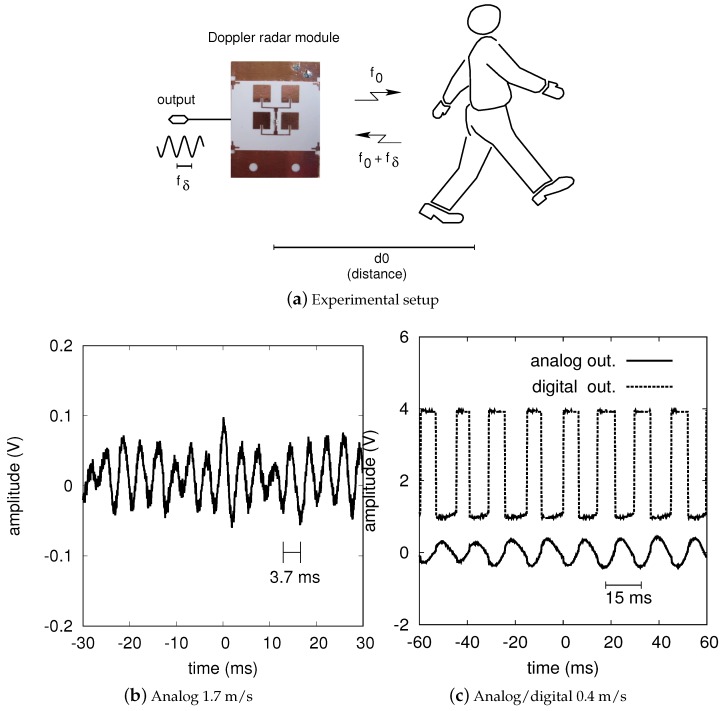
People detection results. (**a**) Experimental setup. (**b**) Analog output corresponding to a person at an 8-m distance with a relative speed of 1.7 m/s. (**c**) Analog and digital outputs associated to a person at a 3-m distance that slowly moves. In the last case the relative speed is of only 0.4 m/s. These signals are measured after the amplification and, possibly, after the triggering stages of the demonstrator (see Figure 4).

The following experiments are aimed at detecting a person in a working environment such as an office [41]. This detection could be useful to activate an intelligent lighting system (lights on only when a person is present) and thus save a significant amount of electric power. The advantage of radars over IR movement sensors is that they are much more sensitive and that they can reliably operate in harsh environments. The scenario is illustrated in Figure 5a.

Figure 5 also reports the Doppler signals obtained from a walking person in different conditions. We started with a person located 8 m from the antenna. Such a distance is in the range of the developed sensor with 67dB of LF gain. The analog output of the radar is shown in Figure 5b. The waveform period is 3.7ms and thus the Doppler frequency is 273Hz. As a result, the radial velocity is 1.7m/s. Observing this graph it is also interesting to note that a low frequency oscillation is superimposed to the 273Hz component. This is due to the swinging nature of the walking motion [13]. In Figure 5c the analog and digital outputs of the radar are recorded instead. In this case a person at a 3-m distance is considered. As the movement begins, the sensor immediately detects it; the pulse period is about 15ms corresponding to a velocity of about 0.4m/s. With other experiments we found that a minimum speed of 50mm/s (Doppler shift equal to 8Hz) is clearly measured up to a distance of 3 m. This demonstrates that the radar can detect very small movements and that it can be reliably used as a motion sensor.

**Figure 6:**
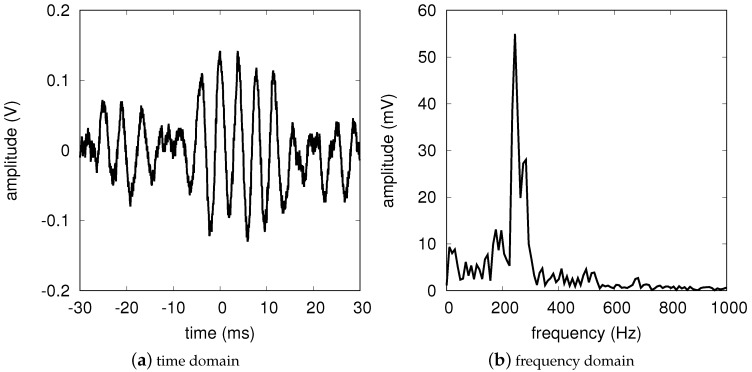
People detection results. (**a**) Time domain output signal corresponding to a person at a 6-m distance with a relative speed of about 1.5 m/s. (**b**) Frequency domain output signal obtained performing the FFT of panel (**a**). The main peak corresponds to a Doppler frequency of 244Hz.

In order to provide a validation of the model stated by Equations (9) and (10), the front-end output was measured for different target distances. The target was again a walking human (European male, height 1.8 m and weight 70 kg) and an indoor experiment was carried out, in a large university laboratory with several metallic closets. First the output voltage of the system in Figure 4 was recorded with the DS1102E digital oscilloscope (Rigol Technologies, Beijing, China) and an FFT was performed on the stored data. As an example, Figure 6, reports the data corresponding to the 6 m experiment. The main FFT peak identifies both the Doppler frequency (*x*-axis) and the output voltage (*y*-axis). The latter value was then divided by the LF gain of the operational amplifier, $A_{LF}=2130$, in order to get the voltage amplitude at the front-end output and thus, according to Equation (1), α. The results of this study are reported in Table 1, column 4, together with the model predictions, column 5. The model values are obtained for γ=38.2mV, as evaluated at the end of Section 2, and assuming a radar cross-section $\sigma =4\;m^{2}$, see [42,43]. With such an assumption one can notice that the experiments are within ±15% with respect to the data predicted by the model.

## 5. Discussion

The state-of-the-art for Doppler radar movement sensors is summarized in Table 2. The design of Droitcour et al. [10] is implemented in 0.25-μm CMOS technology and is applied to cardiopulmonary monitoring, thus confirming that this kind of radar is capable of detecting a very low relative speed. The paper indicates that the measurements are taken a 0.5-m distance. This means that there is margin for a 12-dB gain increase and thus for range doubling.

Two other sensors for low speed detection are reported by Lee et al. [25,27]. These sensors work at 24 GHz and adopt a discrete component electronics on glass-reinforced (RO3003 and FR4, Rogers Corporation, Rogers, CT, USA) or LTCC substrates. The minimum detectable speed reported by the authors ($v_{min}$ in Table 2) is 0.5 and 0.8 mm/s. The measurements are taken a 2-m distance.

The authors of [29,30] instead provide examples of commercial solutions. Both sensors are implemented on an LTCC substrate and are suitable for people detection, robot guidance and automotive applications. The datasheet of the first sensor [30] reports a maximum distance of 30 m. The second sensor [29] is an FMCW radar that, probably can also be used in Doppler mode; the maximum range is 70 m.

Finally, the paper by Alimenti et al. [44], describes a sensor for traffic monitoring. To achieve a long detection range two separate 18-dBi antennas are used (one for the transmitter and one for the receiver), according to a pseudo-monostatic architecture. Furthermore, to achieve a long detection range, the receiver is equipped with a 10-dB low noise amplifier (LNA) and with a 90-dB amplifying chain at low frequency.

This survey highlights that the presented front-end works at a record frequency for cellulose-based circuits. Indeed, no complete circuit on cellulose, similar to that described, can be found in the literature. Furthermore, its performance compares well with those of already published designs based on standard substrates and microelectronic technologies. In particular it is worth noticing that the detection range of the developed front-end is 3 m for the minimum target speed of 50mm/s and about 10 m for a person walking with a speed higher than 1m/s. This range can be further increased if the gain of the OPA chain is increased. To this purpose it should be considered that the output front-end noise (i.e., the noise measured at the output of the 67-dB OPA with the LO switched on) is about 10mV root mean square (RMS).

Concerning fabrication tolerances, we notice that the most sensitive component of the radar front-end is the antenna, since it is constituted by resonant patches. The resolution of the used lithography is of the order of the metal thickness, i.e., around 35μm. With such a resolution we are able to control the resonant frequency of the radiating elements to within 0.6% with respect to the value predicted by computer simulation [40]. Similar deviations were obtained for all the prototypes realized in more than two years of experiments.

The circuit was fabricated in the spring 2014 and, since then, it has been tested many times without showing performance degradation. During the last three years it has been stored in a cabinet, without any particular precautions. On this basis, one can say that the proposed technology is robust enough to operate in indoor environments.

In conclusion, this paper demonstrates that use of circuits on cellulose-based substrates is feasible up to the boundary between microwave and millimeter-waves. In particular, an ultra-low cost Doppler radar front-end operating at 24 GHz is integrated, for the first time, in a multilayer cellulose substrate made up of photo-paper sheets. The front-end is proven to be an excellent motion sensor capable of detecting a walking person up to a distance of about 10 m. A minimum speed of only 50 mm/s was clearly measured, even at a distance of 3 m. The estimated cost of the implemented front-end is less than $2, when produced on a large scale. Finally, the extension of the green approach to the entire system will be the topic of future research; in this perspective the focus is on the implementation of paper-based oscillators capable to operate at 24 GHz. The above results constitute an important step toward the adoption of green electronic processes for disposable sensors and for the electronic hardware in the upcoming IoT era.

## Figures and Tables

**Table 1:** People detection: scale constant α.

$d_{0}$ (m)	$f_{\delta }$ (Hz)	*v* (m/s)	α (μV)	
			Measurements	Model
4	195	1.2	52.6	59.7
6	244	1.5	25.8	26.5
	195	1.2	25.1	
8	273	1.7	16.9	14.9

**Table 2:** State-of-the-art for doppler radar sensors.

Ref.	Technology	$f_{0}$ (GHz)	Antenna Gain (dBi)	$P_{TX}$ (dBm)	Range (m)	$v_{min}$ (mm/s)	Size (mm${}^{2}$)
[10]	0.25-μm CMOS	2.4	8	10	0.5	n.a.	n.a.
[25]	RO3003 and FR4	24	7	15	2	0.5	90×65
[27]	LTCC and FR4	24	n.a.	n.a.	n.a.	0.8	30×30
[29]	LTCC	24	n.a.	20 (*)	70	n.a.	34×21
[30]	LTCC	24	8.6	15 (*)	30	n.a.	25×25
[44]	discrete comp.	24	18	6	300	n.a.	79×79
[32]	cellulose single-layer	24	7	3	n.a.	n.a.	35×28
this work	cellulose multilayer	24	7.4	7	10	50	20×27

(*) Effective isotropic radiated power (EIRP) in dBm.

**Figure A1:**
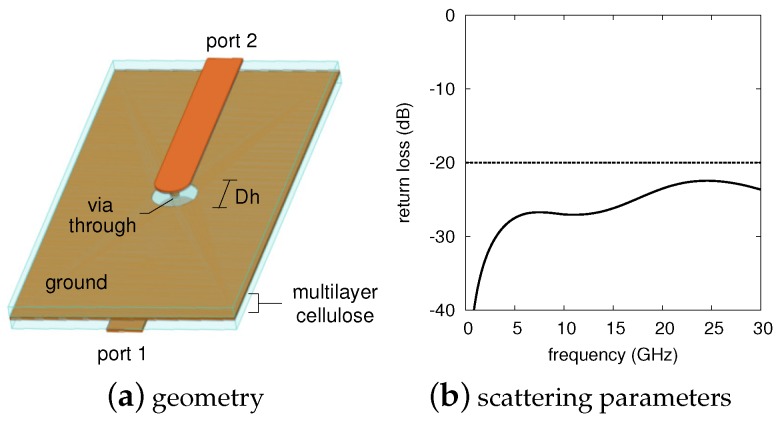
Via-through connection in a multilayer cellulose circuit: structure (**a**) and simulated scattering parameters (**b**). The main geometrical parameters are the hole diameter $D_{h}=1.05\;\mathrm{mm}$, and via diameter of 0.2mm.

**Figure A2:**
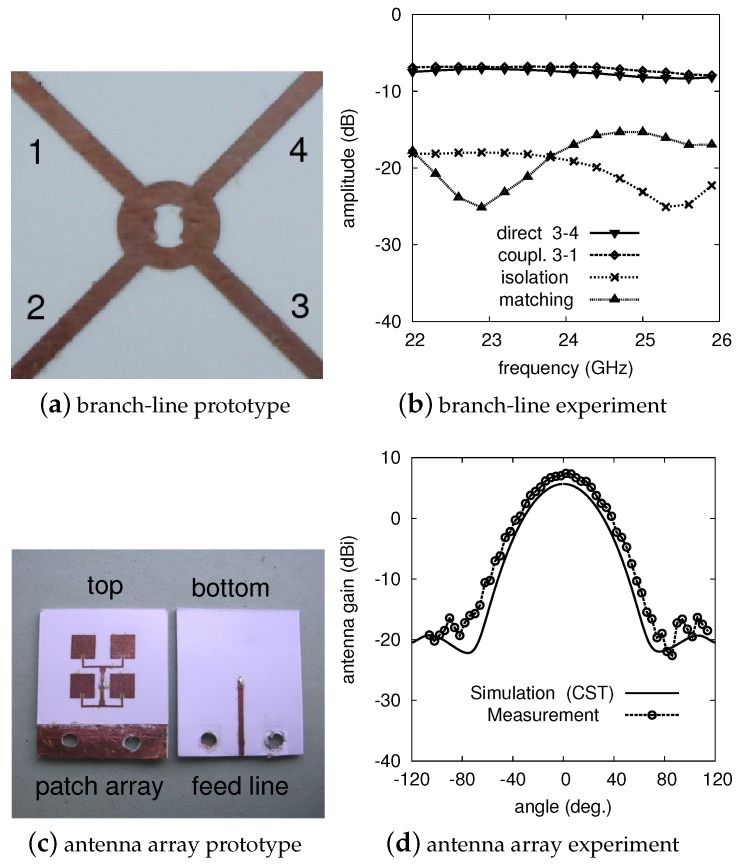
Branch-line coupler and patch antenna array. Branch-line: manufactured prototype (**a**) and experimental results (**b**). The coupler diameter is about 4.4mm. After Reference [39]. Antenna array:manufactured prototype (**c**) and experimental results (**d**). The antenna dimensions are $20\times 20\;{\mathrm{mm}}^{2}$. After Reference [40].

**Figure A3:**
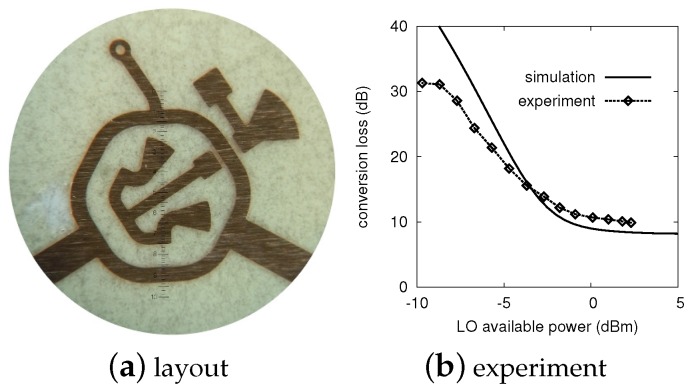
Manufactured layout (**a**) and experimental results (**b**) of the Schottky diode mixer. No components are soldered to the printed circuit board (PCB) shown in (**a**). The active area of the structure is about 4.6mm in diameter. Measured and simulated conversion loss are compared in the plot; these results are obtained by sweeping the LO power between −10dBm and 5dBm with $f_{RF}=24\;\mathrm{GHz}$, $f_{LO}=23.95\;\mathrm{GHz}$, $f_{IF}=50\;\mathrm{MHz}$, and $P_{RF}=-30\;dBm$. After Reference [46].

## Appendix A.

This appendix describes the circuit building-blocks of the cellulose-based Doppler radar front-end, their design, and their experimental characterization.

### Appendix A.1. Via-Through Optimization

One of the key elements in a multilayer circuit is the via-through connection, i.e., the structure that allows metal tracks on different levels to be in galvanic contact. Such a structure is realized using a vertical via that goes from the bottom layer (active side) to the top layer (antenna side) of the front-end board. The central ground plane is discharged in a circular hole to avoid signal shorts.

The via-through shows parasitics of both an inductive (via connection) and capacitive (fringing E-field at microstrip end) nature: at 24GHz these parasitics become critical, thus altering the circuit frequency response significantly. As a consequence, the via-through structure is optimized as follows. First, a simple connection between a 50Ω microstrip line on the bottom layer to another identical line on the top layer is considered as in Figure A1a. Then, the via diameter is selected. In our experimental prototypes the via is implemented by means of a 0.2-mm in the figure) is optimized by means of electromagnetic simulations in order to reduce the return loss of the transition.

Figure A1b reports the via-through return loss simulated with the CST software tool. The optimized hole diameter $D_{h}$, determined through extensive simulations, is equal to 1.05mm. As can be seen from the figure, the obtained reflection coefficient is lower than −20dB in the whole frequency band up to 30GHz. This result shows the theoretical feasibility of multilayer circuits on cellulose.

### Appendix A.2. Branch-Line Coupler

The branch-line coupler is a well-known $90^{\circ }$ hybrid junction widely used at microwave frequencies to split (or divide into two equal parts) an RF signal. Its name comes from the layout of the most common configuration which has two pieces of lines, a quarter-wave long, connected in parallel with two other lines at a mutual distance of λ/4 as well. In the case of the present circuit the ring branch-line coupler is used; the latter one features curved branches rather than straight ones and is more suited to operate at millimeter-wave frequencies. The mathematical description of planar hybrid junctions is reported by several basic text books such as [45] (pp. 379–383).

The present ring coupler is designed with the CST electromagnetic simulator. The fabricated layout is shown in Figure A2a, whereas Figure A2b reports the scattering parameters obtained experimentally. The measured transmission coefficients on the direct (“direct 3-4” in the figure) and coupled (“coupl. 3-1” in the figure) ports are equal to about −7dB. This result is affected by the losses of the lines used to connect the device to the test fixture used in the experiments. De-embedding these losses a coupling factor around −4dB is obtained; such a value is within 10% with the electromagnetic simulations.

### Appendix A.3. Patch Array Antenna

The antenna system is shown in Figure A2c and consists of four rectangular patch elements configured as a 2×2 array. A detailed description of such a component is reported in [40]. The array layout is designed to obtain a broadside radiation while avoiding undesired grating lobes. In order to tune the patch resonance to the operating frequency (24GHz in the present case), the length of each single radiating element is optimized by means of the CST electromagnetic simulator.

The feeding network of the antenna array is formed by three power dividers. The first two power dividers are implemented on the same metal layer as the radiating elements by using two quarter-wave impedance transformers connected to two T-junctions. The third power divider, instead, is obtained adopting the via-through wire which connects the antenna to the bottom metal layer, where the front-end circuitry is placed (see the previous section).

The experiments carried out on the realized prototype show a gain of about 7.4dBi with a radiation efficiency of 35%, where the latter is due to the substrate losses impacting on both the radiating elements and the feeding network. It is worth noticing here that the measured efficiency is in good agreement with the 37% value obtained from simulations (CST). Figure A2d reports the comparison between the measured and the simulated radiation diagram (H-plane cut) at the operating frequency. The agreement between experiments and model is within the measurement accuracy. The obtained half power beam-widths are $55^{\circ }$ in the E-plane and $48^{\circ }$ in the H-plane. Moreover, the antenna has an input reflection coefficient of about −29dB at the center frequency and an operating bandwidth of 540MHz (reflection coefficient lower than −20dB).

### Appendix A.4. Single-Balanced Diode Mixer

The mixer is a key building block in microwave sensors and telecommunication front-ends, and allows for the frequency conversion from the RF to the intermediate frequency (IF), which is performed by means of the LO signal. In Doppler radars it is used to detect the small frequency variations between the transmitted and received signals.

The mixer adopted in our sensor is described in [46] and the manufactured layout is shown in Figure A3a. It uses a single-balanced diode topology and a ring structure. Only three lumped components are needed, namely: two low-barrier Schottky diodes and a 0Ω resistor, used as a jumper. A miniaturization of the mixer is obtained by placing the two diodes and the two quarter-wave open-stubs (used to short-circuit the RF signals) inside the ring. Such a ring has a perimeter of 3λ/2 and a characteristic impedance of 70.7Ω. Other stubs, outside the ring, are used to match the RF and LO ports to 50Ω and to provide a further isolation between IF and RF/LO ports. The design is performed with the ADS Harmonic Balance (HB) simulator.

The aforementioned mixer is intended for zero- or low-IF operation and works at 24GHz, with a bandwidth of ±150MHz. The conversion loss, defined as the difference between the (available) RF and the (delivered) IF powers in dBm, is used to evaluate the circuit performance. The experimental results are reported in Figure A3b and are obtained by applying a 24-GHz RF and a 23.95-GHz LO signal respectively to the mixer ports. As a consequence, an IF signal at 50MHz is obtained: the measured conversion loss in such a condition is around 10dB. The LO to RF isolation at center frequency is better than 35dB.

The front-end gain, $G_{RF}$ in Figure 1, can be estimated by summing the mixer conversion losses with the branch-line coupler insertion losses and changing the sign to the result: $G_{RF}\approx -\left(10+4\right)=-14\;\mathrm{dB}$, equivalent to about 1/25 in linear scale.
